# Cancer gene therapy clinical trials: lessons for the future

**DOI:** 10.1054/bjoc.2001.2129

**Published:** 2001-11

**Authors:** E Galanis, S Russell

**Affiliations:** Mayo Clinic, Rochester, MN, USA

**Keywords:** gene therapy, cancer, clinical trials


*British Journal of Cancer* (2001) **85**(10), 1432–1436
© 2001 Cancer Research Campaign
doi: 10.1054/ bjoc.2001.2129, available online at http://www.idealibrary.com on IDEAL

http://www.bjcancer.com

Review

### Cancer gene therapy clinical trials: lessons for the future

**E Galanis and S Russell**

Mayo Clinic, Rochester, MN, USA

**Keywords**: gene therapy; cancer; clinical trials

### BACKGROUND

The concept of gene therapy evolved from the initial observation that certain diseases are caused by the inheritance of a single functionally defective gene. Theoretically, diseases caused by a genetic defect could be treated and potentially cured by the insertion and expression of a normal copy of the mutant or deleted gene in host cells. The idea of gene replacement therapy represents the basic framework behind therapeutic approaches to monogenic disease. Following early gene therapy efforts targeting inherited diseases such as adenosine deaminase deficiency and cystic fibrosis, it became obvious that the limited efficiency of gene transfer that could be accomplished with the existing vectors, would make treatment of monogenic hereditary diseases by using gene transfer approaches extremely challenging. As a result, the evolution of clinical gene transfer efforts has taken a somewhat unexpected course.

To date, almost two-thirds of the gene therapy clinical trials (more than 300 trials worldwide) are focusing on cancer. One important motivation that underlies the shift has been the evolution of cancer gene therapy approaches that are less affected by the technical limitations complicating treatment of inherited genetic diseases. These approaches attempt to increase tumour cell immunogenicity and/or enhance killing via gene replacement, or suicide gene transfer and, opposite to the manipulations directed toward overcoming metabolic disorders, do not require sustained and tightly regulated gene expression in target cells (Roth and Cristiano, 1997; Anklesaria, 2000).

Nevertheless, since the development of the first cancer gene therapy clinical trials in the early 1990s, one major criticism has focused on the concern that more scientific proof was necessary prior to gene transfer approaches being introduced to the clinic (Crystal, 1995). On the other hand, the suboptimal ability of animal models to accurately predict safety and efficacy of gene transfer in humans made clinical trials necessary in order to first and foremost establish safety and vector kinetics, with efficacy being a secondary endpoint.

During the last 2 years, an impressive amount of public attention has been focused on gene therapy as a result of two events. Although neither of them occurred in cancer gene therapy clinical trials, they definitely have affected the evolution of clinical standards in the field of cancer gene therapy.

*Correspondence to:* E Galanis

The first event was the death of an adult patient with ornithine transcarbamylase (OTC deficiency) in a clinical trial at the University of Pennsylvania. In this study, either female carriers or males with a mild form of the disease were treated via the hepatic artery with escalating doses of a non-replicating adenovirus, containing the gene for ornithine transcarbamylase. An 18-year-old patient in the sixth (highest) dose cohort who received an approximate total of $4 \times 10^{13}$ viral particles died 4 days after treatment. Initial investigation of the events leading to the patient's death concluded that the probable cause of death was acute respiratory distress syndrome, resulting from an inflammatory response possibly secondary to activation of innate immunity (Marshall, 1999). This event resulted in media frenzy and an outpouring of negative commentary in the lay press, regarding the potential risks associated with gene transfer, the apparent oversight, and the suboptimal requirements for recording and reporting serious adverse events in gene therapy trials. Although a good deal of the discussion was based on misunderstanding or misinformation on the incidence of adverse events in phase I clinical studies and the already existing reporting requirements, still some critical lessons have been emphasized: rules and guidelines for conducting preclinical experiments, clinical trials, and quality assurance testing of materials, including gene delivery vectors, should be reinforced. In addition, as the field of gene therapy evolves, there is a major need to compose data regarding efficiency of gene delivery, safety and toxicity in accessible databases so that this already accumulated information can be translated into useful knowledge for all investigators.

On the positive side, however, the first unequivocal demonstration of clinical gene transfer efficacy was achieved by ex vivo treatment of 2 infants, using a retrovirus to deliver the γC cytokine receptor gene to treat one of the most severe immunodeficiencies, the SCID-X1 disease (Cavazzana-Calvo et al, 2000). This success is the first significant demonstration of long-term clinical benefit (lasting nearly one year at the time of the original report) that resulted from gene delivery in human trials. Despite the fact that the actual duration of the clinical benefit is not clear yet, this result has generated great optimism regarding the eventual realization of clinical benefits from gene delivery.

This review will expand on lessons learned during the last 10 years from gene transfer clinical trials in the treatment of cancer, mainly focusing on safety and vector kinetics. Discussion on efficacy will be limited to pertinent demonstrations of general principles rather than an exhausting description of detailed results.

### SAFETY IN CANCER GENE THERAPY CLINICAL TRIALS

Several clinical trials during the last decade have proven the safety of intratumoural administration of a variety of vectors, both replicating and non-replicating for different therapeutic transgenes; among them retroviral, adenoviral vectors, vaccinia virus, and DNA/liposome complexes. Sites of administration include skin and subcutaneous tumour deposits, lymph nodes (Stopeck et al, 1997), the central nervous system (Ram et al, 1997; Klatzmann et al, 1998; Packer et al, 2000), prostate gland (Herman et al, 1999; Sweeney and Pisters, 2000), and a variety of other intra-abdominal and thoracic visceral organs such as liver and lungs (Rubin et al, 1997; Galanis et al, 1999). Most of these injections were performed under direct CT or ultrasound guidance, while bronchoscopic administration has also been employed for endobronchial lesions (Roth et al, 1996) and administration through endoscopic ultrasound for pancreatic lesions (Mulvihill et al, 2001).

Toxicity in these early studies appears to be related mainly to the injection procedure with up to 50% of the patients experiencing mild pain and discomfort at the injection site when gene transfer is performed under CT or US guidance (Galanis et al, 1999). The incidence of pneumothorax after CT-guided or bronchoscopic delivery of an adenoviral vector encoding p53 in patients with advanced NSCL cancer varied from 7% (6/84 injections) (Swisher et al, 1999) to 21% (13/61 injections) (Swisher et al, 2000) in different clinical trials.

Vector-related toxicity was dependent, as expected, on the type of the vector, the dose, and the site of administration. For example, intracranial administration of retrovirus-producing fibroblasts into the resection cavity of patients with recurrent glioblastoma multiforme has resulted in subarachnoid haemorrhage and aseptic ventriculitis in 2/12 patients (Klatzmann et al, 1998). Fever and chills appeared to be common side effects after administration of non-replicating adenoviruses such as an adenovirus encoding the *p53* gene into locally recurrent head and neck tumours (Clayman et al, 1998) or in the prostate gland (Sweeney and Pisters, 2000) of patients with local recurrence after radiation therapy. They were related to the viral dose and more common when doses $> 10^{10}$ pfu were employed. Intratumoural injections of recombinant vaccinia virus encoding GMCSF in patients with dermal or subcutaneous metastasis from cutaneous melanoma resulted in development of mild flu-like symptoms that resolved within 24 hours and local inflammation with pustule formation when doses $> 10^7$ pfu were employed (Mastrangelo et al, 1999).

Certain administration sites such as the peritoneal cavity impose additional challenges. Intraperitoneal administration of viral vectors can result in sterile peritonitis; 3/12 patients developed this reaction as evidenced by patient discomfort, fever, increased peritoneal fluid cell counts, and negative bacterial cultures, after receiving a retroviral vector encoding for *BRCA1* (up to a total dose of $10^{10}$ viral particles) (Tait et al, 1997). Similarly, inflammatory infiltrate was observed after intrapleural administration of an adenoviral vector encoding HSV-tk (Molnar-Kimber et al, 1998).

Less frequently, side effects have been attributed to therapeutic transgenes such as mild flu-like symptoms seen after intratumoural administration of IL-2/cDNA/DMRIE/DOPE lipid complex (Galanis et al, 1999) and to the prodrug used in suicide approaches (i.e. LFT elevation as a result of ganciclovir) (Sterman et al, 1998). Of note, large doses of plasmid DNA (up to 4 mg per dose in a repeat administration schedule) appear to be well tolerated, without evidence of development of anti-DNA antibodies (Daniels and Galanis, 2001).

Nevertheless, advanced cancer is predominately a systemic disease. Therefore, with the exception of immunotherapeutic approaches attempting in situ vaccination, as well as gene delivery to treat certain tumour types such as brain tumours and ovarian cancer that rarely metastasize, it soon became obvious that improved gene transfer efficiency and systemic administration of gene transfer vectors would be necessary in order to improve the clinical applicability of gene transfer technology. For this to happen, a dogma of the early clinical days of gene transfer opposed to the use of replicating vectors had to be overcome. In addition, the importance of delivering gene transfer vectors systemically (intravenously or intra-arterially), became obvious (Russell, 1994).

Most clinical experience with replicating viruses has been gained with the E1B attenuated adenovirus ONYX-015 (Bishoff et al, 1996) that appears to selectively, but not exclusively (Goodrum and Ornelles, 1998; Rothman et al, 1998), replicate in cells with malfunctioning p53. Clinical trials with this agent have been completed in patients with head and neck cancer (Khuri et al, 2000), pancreas (Mulvihill et al, 2001), ovarian cancer (Vasey et al, 2000), and GI malignancies metastatic to the liver (Bergland et al, 1998). Other replicating viruses recently introduced to the clinic include the provisionally replicating adenovirus CN207 in which the expression cassette is driven by PSA promoter/enhancer elements and, therefore, it can selectively replicate in prostate tissue (Rodriguez et al, 1997). A recently completed phase I/II trial in patients with locally recurrent prostate cancer, after failure of radiation, showed that doses of up to $10^{13}$ particles of the CN207, administered using brachytherapy techniques, appeared to be safe, although biochemical (PSA) responses were observed in a minority of patients (Simons et al, 2000). Additionally, a double mutant herpes simplex virus engineered with deletion of both copies of $\gamma 34.5$ gene and a lacZ insertion disabling the U39 gene (encoding the large subunit of the viral ribonucleotide reductase), has been administered stereotactically in patients with recurrent gliomas at doses up to $3 \times 10^9$ pfu without any significant toxicity, including encephalitis, being encountered (Market et al, 2000). Other replicating viruses currently in clinical trials include the reovirus, a virus that replicates in malignant cells with an activation in the ras signaling pathway (Coffey et al, 1998) and the animal pathogen Newcastle disease virus (Pecora et al, 2001).

As it pertains to systemic administration of viral vectors, the clinical work has been focused mainly on adenoviruses, both replicating and non-replicating. Hepatic artery administration of a non-replicating adenovirus encoding the tumour suppressor gene *p53* showed that very high adenoviral doses of approximately $7.5 \times 10^{13}$ particles could be associated with hypotension (Venook et al, 1998). In contrast, lower doses of both the non-replicating p53 adenovirus (approximately $2.5 \times 10^{13}$ particles) and $2 \times 10^{12}$ particles of the replicating adenovirus ONYX-015, administered through the hepatic artery, were well tolerated (Reid et al, 2000). The most common side effects associated with the administration of the ONYX virus include mild constitutional symptoms and reversible LFT elevation. Intravenous administration of ONYX-015 in doses up to $2 \times 10^{12}$ viral particles was also well tolerated except for mild to moderate constitutional symptoms (Nemunaitis, personal communication).

© 2001 Cancer Research Campaign

### VECTOR KINETICS IN CANCER GENE THERAPY CLINICAL TRIALS

In a minority of reported trials, not only issues pertaining to safety, but also kinetics of the vectors employed, were addressed. A phase I/II trial of intratumoural administration of a p53 encoding adenovirus (Adp53) in patients with recurrent head and neck cancer (Clayman et al, 1998) showed that Adp53 DNA was detected in blood by PCR by 30 minutes after Adp53 injection and gradually eliminated over the next 48 hours. Cytopathic effect (CPE) assays performed in patients treated at $3 \times 10^{10}$ and $10^{11}$ pfu (the highest 2-dose levels) showed that viable Adp53 was present in blood at the highest levels 30 minutes after intratumoural injections, decreased at a rate of 2 – 4 orders of magnitude by 90 minutes and further decreased to very low or undetectable titres by 24 hours to be completely eliminated by 48 hours after injection. Ad p53 was detected in the urine from some patients who received doses of $3 \times 10^9$ pfu or greater and was present in urine from all patients who received doses of $3 \times 10^{10}$ pfu or greater. Adp53 detection in the urine started within one day of the beginning of p53 injections and was detected through repeat treatment courses. The highest titre detected in the urine was $10^6$ pfu/0.5 ml. Urine was free of Adp53 within 3 – 17 days of the last Adp53 injection.

Adp53 was also detected in the sputum and/or saliva samples of 6 high-dose patients tested. As with urine samples, Adp53 was detected within one day of injection, was present for several days after the last injection of the virus, and was cleared to background levels within 7 days. Similarly, an HSV-tK adenovirus was detected in a dose-dependent manner in the urine of all patients after intraprostatic administration (Herman et al, 1999).

When the replicating adenovirus ONYX-015 was administered intrahepatically, 2 peaks of viral titres were detected by PCR in the peripheral blood; one 30 minutes after intra-arterial administration; and the second, 3 days later, consistent with viral replication (Reid et al, 2000). Of note, in all adenoviral trials, the level of neutralizing antibodies appeared to increase significantly after administration of the virus within 3 – 4 weeks. Antibody titre however, did not appear to correlate with adenoviral dose or course of treatment and did not prevent the expression of therapeutic transgene after intratumoural administration or viral replication after intra-arterial administration (Clayman et al, 1998; Reid et al, 2000).

A significant public health issue relates to the exposure of health-care providers and family members to potentially harmful viral vectors. This has been only minimally or inadequately addressed in most clinical trials. In one study of intratumoural administration of adenoviral vectors, 2 health providers with the greatest risk of exposure were tested. No elevation of neutralizing antibodies was observed in their serum, and neither serum nor urine contained infectious p53 particles or Adp53 DNA (Clayman et al, 1998).

### EFFICACY

There are occasional reports of efficacy in cancer gene therapy clinical trials employing a variety of approaches including immunotherapy, tumour suppressor gene reconstitution, and prodrug activation therapy.

Examples of clinical efficacy mediated through immunotherapeutic gene transfer approaches include partial responses observed in 2/14 renal cell carcinoma and 1/16 melanoma patients after intratumoural administration of *IL-2* gene DMRIE/DOPE lipid complex (Galanis et al, 1999), responses of the injected lesions in up to 20% of the patients when the *HLA-B7* gene in combination with the cationic lipid complex DMRIE/DOPE was administered intratumourally in melanoma patients (Hersch and Stopeck, 1997), and a partial response achieved in a patient with metastatic renal cell carcinoma to the lungs when an autologous vaccine consisting of patients' own tumour cells transduced with a retroviral vector encoding GMCSF was employed (Simons et al, 1997). Generally, immunotherapy approaches are less affected by the limited efficacy of gene transfer as compared to other gene transfer strategies, since transfection or transduction of a relatively small percentage of cancer cells may be still adequate in order to elicit immunologic response.

Using a 'gene-replacement' approach, transient local disease control has been observed after bronchoscopic administration of a p53 retroviral vector in 3/8 patients (Roth et al, 1996). In a subsequent phase I study of bronchoscopic or CT-guided intratumoural injection of a non-replicating adenovirus encoding p53, 2/28 patients exhibited partial responses (Swisher et al, 1999). Similar results were obtained with the use of p53 adenovirus in patients with head and neck cancer (Clayman et al, 1998). Of note, the presence of circulating neutralizing antibodies did not preclude gene transfer or antitumour activity, a principle that has been demonstrated not only for intratumoural, but also for intrapleural (Molnar-Kimber et al, 1998) and intrahepatic administration of adenoviral vectors (Venook et al, 1998).

As it pertains to prodrug activation therapy, intracranial stereotactic administration of retroviral vector producer cells (VPCs) producing a Maloney Leukemia virus vector coding for the *HSV-tk* gene, followed by treatment by ganciclovir, resulted in 5 objective responses out of 15 patients with recurrent brain tumours who had the VPCs introduced stereotactically (Ram et al, 1997). This type of encouraging data led to a large multinational phase III study in newly diagnosed patients with glioblastoma multiforme who, after surgery, were randomized to radiotherapy versus HSV-tk/ganciclovir gene therapy, followed by radiotherapy. No significant differences were observed between the 2 treatment arms, possibly pointing to the prematurity of the attempt (Rainov, 2000).

Although initially gene transfer has been envisioned by many as being able to eradicate cancer as a single modality, a principle that has recently emerged is the value of combining gene transfer with traditional anticancer modalities such as chemotherapy and radiation therapy. That has been nicely demonstrated for the replicating adenovirus ONYX-015 in combination with 5-FU chemotherapy (Heise et al, 1997) and confirmed in a clinical trial in head and neck patients where viral monotherapy led to objective responses in only 15% of the patients as compared to 60% of the patients when this treatment was combined with 5-FU/cisplatin chemotherapy (Khuri et al, 2000). Based on historic controls, chemotherapy alone in this setting had only a 35% chance of response. This principle is further investigated in an ongoing phase III study, randomizing patients who have failed radiation therapy for recurrent head and neck cancer between treatment with ONYX-015 in combination with 5-FU/cisplatin versus 5-FU/cisplatin alone. The mechanism of synergy has not been completely elucidated. It is possible that ONYX-015 is able to sensitize infected and uninfected cells to chemotherapy-induced cell death. EIA gene expression is an important chemosensitizer, and this effect is independent of p53 in some models. As ONYX-015 expresses E1A in both p53-deficient and p53-functional cancer

 *© 2001 Cancer Research Campaign*

cells, this mechanism may account for the chemosensitization of both tumour types in vitro. However, E1A expression does not chemosensitize normal non-transformed cells. In addition, adenovirus-induced cytokines such as tumour necrosis factors can act as important chemosensitizers. Elucidation of the mechanisms involved in the chemosensitization may allow enhancement of this effect in future trials.

Similarly, intratumoural administration of the replication deficient p53 adenovirus, along with cisplatin chemotherapy, appears to chemosensitize a variety of tumour cell lines (Nielsen et al, 1997) including lung cancer cells and tumours in vivo to the effect of chemotherapy: 2/24 patients with refractory platinum non-small-cell lung cancer exhibited partial responses and stable disease was seen in 17 patients (Nemunaitis et al, 2000). This is presumably the result of restoration of an apoptotic mechanism of cell death in these cancer cells; in situ nick-end labelling assay demonstrated increase in apoptosis in 79% of the patients.

Combination of gene transfer with radiation therapy is also a subject of ongoing evaluation. Based on encouraging preclinical work, a phase I/II trial of radiation therapy in combination with 3 biweekly intratumoural injections of Ad p53 in patients with locoregionally advanced non-small-cell lung cancer, achieved one year progression-free survival of 45.5%, which is superior to historic controls (Swisher et al, 2000). Similarly, the conditionally replicating adenovirus ONYX-015 had a synergistic effect when combined with radiation therapy in radiation-resistant glioma xenografts (Georger et al, 2000).

In summary, a significant amount of information has emerged from cancer gene therapy clinical trials during the last 10 years. As a result of this experience, when vector systems previously used in the clinic are employed, demonstration of safety is no longer an adequate justification for clinical trials in the absence of preclinical data supporting also the efficacy of the approach. In addition, for viruses already used in the clinic, it is questionable if the traditional phase I design is an appropriate means of determination of the phase II dose. It appears that for both adenoviruses and retroviruses, the maximum number of infectious particles delivered intratumourally is only limited by the titres that can be produced. Different rules apply, however, when intra-arterial or intravenous vector administration is contemplated, or when novel replicating viral vectors are introduced to the clinic. In these settings, safety has to be convincingly demonstrated.

Correlative endpoints, including evaluation of expression of the transferred genes, immunologic response to transgenes and vectors (when appropriate), vector kinetics and assessment of viral replication, if replicating vectors are employed, are crucial in order to validate the clinical utility of a given approach, in combination with more traditional endpoints such as assessment of safety and efficacy.

In addition, strict reporting requirements will have to be followed in order to ensure the safety of patients participating in clinical gene transfer studies. The National Institute of Health in the United States is in the process of implementing additional safeguards to better oversee safety aspects of clinical gene therapy trials. The Department of Health and Human Services reorganized the Office for Human Research Trials. A National Human Research Protection Advisory Committee was established and the Food and Drug Administration (FDA) created the Office for Human Research Trials (Ready, 2001). While it remains an ongoing challenge, there is gradually increasing hope that new developments in the field of gene transfer, including vector targeting, new vector systems, replicating vectors, and novel transgenes, along with constructive use of lessons learned in the recent past, will allow the safe and efficient incorporation of gene transfer in the treatment of cancer.

### ACKNOWLEDGEMENTS

Supported in part by NCI CA-84388 (EG).

### REFERENCES

Anklesaria P (2000) Gene therapy: A molecular approach to cancer treatment. *Curr Opin Mol Ther* **2**(4): 426–432

Bergsland E, Mani S, Kirn D, Fell S, Heise C, Maack C, Venook A and Warren R (1998) Intratumoral injection of ONYX-015 for gastrointestinal tumors metastatic to the liver: A phase I trial. *Proc Am Soc Clin Oncol* **17**: 211a

Bischoff JR, Kirn DH, Williams A, Heise C, Horn S, Muna M, Ng L, Nye JA, Sampson-Johannes A, Fattaey A and McCormick F (1996) An adenovirus mutant that replicates selectively in p53-deficient human tumor cells. *Science* **274**: 373–376

Cavazzana-Calvo M, Hacein-Bey S, de Saint Basile G, Gross F, Yvon E, Nusbaum P, Selz F, Hue C, Certain S, Casanova J-L, Bousso P, Le Deist F and Fischer A (2000) Gene therapy of human severe combined immunodeficiency (SCID)-XI disease. *Science* **288**(5466): 669–672

Clayman GL, El-Naggar AK, Lippman SM, Henderson YC, Frederick M, Merritt JA, Zumstein LA, Timmons TM, Liu T-J, Ginsberg L, Roth JA, Hong WK, Bruso P and Goepfert H (1998) Adeovirus-mediated p53 gene transfer in patients with advanced recurrent head and neck squamous cell carcinoma. *J Clin Oncol* **16**(6): 2221–2232

Coffey MC, Strong JE, Forsyth PA and Lee PW (1998) Reovirus therapy of tumors with activated Ras pathway. *Science* **282**: 1332–1334

Crystal R (1995) Transfer of genes to humans: early lessons and obstacles to success. *Science* **270**: 404–410

Daniels GA and Galanis E (2001) Immunotherapy of renal cell carcinoma by intratumoral administration of an IL-2 cDNA/DMRIE/DOPE lipid complex. *Curr Opin Mol Ther* **3**(1): 70–76

Galanis E, Hersh EM, Stopeck AT, Gonzalez R, Burch P, Spier C, Akporiaye ET, Rinehart JJ, Edmonson J, Sobol RE, Forscher C, Sondak VK, Lewis BD, Unger EC, O'Driscoll M, Selk L and Rubin J (1999) Immunotherapy of advanced malignancy by direct gene transfer of an interleukin-2 DNA/DMRIE/DOPE lipid complex: Phase I/II experience. *J Clin Oncol* **17**(10): 3313–3323

Georger B, Grill J, Opolon P, Terrier-Lacombe M, Morizet J, Aubert G, Kirn DH and Vassal G (2001) ONYX-015, an E1B-55kD deleted adenovirus, potentiates radiation therapy in human malignant glioma xenografts. *Proc Am Soc Clin Oncol* **20**: 53a

Goodrum FD and Ornelles DA (1998) p53 status does not determine outcome of E1B 55-kilodalton mutant adenovirus lytic infection. *J Virol* **72**: 9479–9490

Heise C, Sampson-Johannes A, Williams A, McCormick F, Von Hoff DD and Kirn DH (1997) ONYX-015, an E1B gene-attenuated adenovirus, causes tumor-specific cytolysis and antitumoral efficacy that can be augmented by standard chemotherapeutic agents. *Nat Med* **3**: 639–645

Herman JR, Adler HL, Aguilar-Cordova E, Rojas-Martinez A, Woo S, Timme TL, Wheeler TM, Thompson TC and Scardino PT (1999) In situ gene therapy for adenocarcinoma of the prostate: A phase I clinical trial. *Hum Gene Ther* **10**: 1239–1249

Hersch EM and Stopeck AT (1997) Advances in the biological therapy and gene therapy of malignant disease. *Clin Cancer Res* **12**Pt2: 2623–2629

Khuri FR, Nemunaitis J, Ganly I, Arseneau J, Tannock IF, Romel L, Gore M, Ironside J, MacDougall RH, Heise C, Randlev B, Gillenwater AM, Bruso P, Kaye SB, Hong WK and Kirn DH (2000) A controlled trial of intratumoral ONYX-015, a selectively-replicating adenovirus, in combination with cisplatin and 5-fluorouracil in patients with recurrent head and neck cancer. *Nat Med* **6**(8): 879–885

Klatzmann D, Valery CA, Bensimon G, Marro B, Boyer O, Mokhtari K, Diquet B, Salzmann JL and Philipporn J (1998) A phase I/II study of herpes simplex virus type I thymidine kinase 'suicide' gene therapy for recurrent glioblastoma. *Hum Gene Ther* **9**: 2595–2604

Markert JM, Medlock MD, Rabkin SD, Gillespie GY, Todo T, Hunter WD, Palmer CA, Feigenbaum F, Tornatore C, Tufaro F and Martuza RL (2000) Conditionally replicating herpes simplex virus mutant, G207 for the treatment of malignant glioma: Results of a phase I trial. *Gene Ther* **7**: 867–874

Marshall E (1999) Clinical trials: Gene therapy death prompts review of adenovirus vector. *Science* **286**: 2244–2245

*© 2001 Cancer Research Campaign*

Mastrangelo MJ, Maguire HC, Eisenlohr LC, Laughlin CE, Monken CE, McCue PA, Kovatich AJ and Lattime EC (1999) Intratumoral recombinant GM-CSF-encoding virus as gene therapy in patients with cutaneous melanoma. *Cancer Gene Ther* **6**: 409–422

Molnar-Kimber KL, Sterman DH, Chang M, Kang EH, ElBash M, Lanuti M, Elshami A, Gelfand K, Wilson JM, Kaiser LR and Albelda SM (1998) Impact of preexisting and induced humoral and cellular immune responses in an adenovirus-based gene therapy phase I clinical trial for localized mesothelioma. *Hum Gene Ther* **9**(14): 2121–2133

Mulvihill SJ, Warren R, Venook A, Adler A, Randlev B, Heise C and Kirn D (2001) Safety and feasibility of injection with an E1B-55 kDa gene-deleted, replication-selective adenovirus (ONYX-015) into primary carcinomas of the pancreas: A phase I trial. *Gene Ther* **8**(4): 308–315

Nemunaitis J, Swisher SG, Timmons T, Connors D, Mack M, Doerksen L, Weill D, Wait J, Lawrence DD, Kemp BL, Fossella F, Glisson BS, Hong WK, Khuri FR, Kurie JM, Lee JJ, Lee JS, Nguyen DM, Nesbitt JC, Perez-Soler R, Pisters KM, Putnam JB, Richli WR, Shin DM, Walsh GL, Merritt J and Roth J (2000) Adenovirus mediated p53 gene transfer in sequence with cisplatin to tumors of patients with non-small-cell lung cancer. *J Clin Oncol* **18**: 609–622

Nielsen LL, Lipari P, Dell J, Gurnani M and Hajian G (1998) Adenovirus-mediated *p53* gene therapy and paclitaxel have synergistic efficacy in models of human head and neck, ovarian, prostate, and breast cancer. *Clin Cancer Res* **4**: 835–846

Pecora AL, Rizvi N, Cohen GI, Meropol NJ, Sterman D, Marshall J and Lorence RM (2001) An intravenous phase I trial of a replication-competent virus, PV701, in the treatment of patients with advanced solid cancers. *Proc Am Soc Clin Oncol* **20**: 253a

Rainov NG (2000) A phase III clinical evaluation of herpes simplex virus type 1 thymidine kinase and ganciclovir gene therapy as an adjuvant to surgical resection and radiation in adults with previously untreated glioblastoma multiforme. *Hum Gene Ther* **11**: 2389–2401

Ram Z, Culver KW, Oshiro EM, Viola JJ, DeVroom HL, Otto E, Long Z, Chiang Y, McGarrity GJ, Muul LM, Blaese RM and Oldfield EH (1997) Therapy of malignant brain tumors by intratumoral implantation of retroviral vector-producing cells. *Nature Med* **3**: 1354–1361

Ready T (2001) RAC tries to regain foothold. *Nature Med* **7**(9): 983

Reid T, Galanis E, Abbruzzese J, Sze D, Romel L, Hatfield M, Rubin J and Kirn D (2000) Hepatic artery infusion of Onyx-015, a selectively-replicating adenovirus, in combination with 5-FU/leucovorin for gastrointestinal carcinoma metastatic to the liver: a phase I/II trial. *Proc Am Soc Clin Oncol* **19**: 246a

Rodriguez R, Schuur ER, Lim HY, Henderson GA, Simons JW and Henderson DR (1997) Prostate attenuated replication competent adenovirus (ARCA) CN706: A selective cytotoxic for prostate-specific antigen-positive prostate cancer cells. *Cancer Res* **57**: 2559–2563

Roth JA and Cristiano RJ (1997) Gene therapy for cancer: What have we done and where are we going? *J Natl Cancer Inst* **89**: 21–39

Roth JA, Nguyen D and Lawrence DD (1996) Retrovirus-mediated wild-type p53 gene transfer to tumors of patients with lung cancer. *Nat Med* **2**: 985–991

Rothman T, Hengstermann A, Whitaker NJ, Scheffner M and zur Hausen H (1998) Replication of ONYX-015, a potential anticancer adenovirus, is independent of p53 status in tumor cells. *J Virol* **72**: 9470–9478

Rubin J, Galanis E, Pitot HC, Richardson RL, Burch PA, Charboneau JW, Reading CC, Lewis BD, Stahl S, Akporiaye ET and Harris DT (1997) Phase I study of immunotherapy of hepatic metastases of colorectal carcinoma by direct gene transfer of an allogeneic histocompatibility antigen, HLA-B7B7. *Gene Ther* **4**: 419–425

Russell SJ (1994) Replicating vectors for gene therapy of cancer: Risks, limitations and prospects. *Eur J Cancer* **30**A: 1165–1171

Schuler M, Herrmann R, DeGreve JL, Stewart AK, Gatzemeier U, Stewart DJ, Laufman L, Gralla R, Kuball J, Buhl R, Heussel CP, Kommoss F, Perruchoud AP, Shepherd FA, Fritz MA, Horowitz JA, Huber C and Rochlitz (2001) Adenovirus-mediated wild-type p53 gene transfer in patients receiving chemotherapy for advanced non-small-cell lung cancer: results of a multicenter phase II study. *J Clin Oncol* **19**(6): 1750–1758

Simons JW, Jaffee EM, Weber CE, Levitsky HI, Nelson WG, Carducci MA, Lazenby AJ, Cohen LK, Finn CC, Clift SM, Hauda KM, Beck LA, Leiferman KM, Owens AH, Jr., Piantadosi S, Dranoff G, Mulligan RC, Pardoll DM and Marshall FF (1997) Bioactivity of autologous irradiated renal cell carcinoma vaccines generated by ex vivo granulocyte-macrophage colony-stimulating factor gene transfer. *Cancer Res* **57**: 1537–1546

Simons JW, Mikhak B, Van Der Poel HG, DeMarzo A, Rodriguez R, Goemann MA, Nelson WG, Li S, Detorie N, Hamper UM, Ramakrishna N and DeWeese TL (2000) Molecular and clinical activity of CN706, a PSA-selective oncolytic AD5 vector in a phase I trial in locally recurrent prostate cancer following radiation therapy. *Mol Ther* **1**(5): S144–S145

Sterman DH, Treat J, Litzky LA, Amin KM, Coonrod L, Molnar-Kimber K, Recio A, Know L, Wilson JM, Albelda SM and Kaiser LR (1998) Adenoviruis-mediated herpes simplex virus thymidine kinase/ganciclovir gene therapy in patients with localized malignancy: Results of a phase I clinical trial in malignant mesothelioma. *Hum Gene Ther* **9**: 1083–1092

Stopeck AT, Hersh EM, Akporiaye ET, Harris DT, Grogan T, Unger E, Warneke J, Schluter SF and Stahl S (1997) Phase I study of direct gene transfer of an allogeneic histocompatibility antigen, HLA-B7, in patients with metastatic melanoma. *J Clin Oncol* **15**: 341–349

Sweeney P and Pisters LL (2000) Ad5CMVp53 gene therapy for locally advanced prostate cancer – where do we stand? *World J Urol* **18**: 121–124

Swisher SG, Roth JA, Nemunaitis J, Lawrence DD, Kemp BL, Carrasco CH, Connors DG, El-Naggar AK, Fossella F, Glisson BS, Hong WK, Khuri FR, Kurie JM, Lee JJ, Lee JS, Mack M, Merritt JA, Nguyen DM, Nesbitt JC, Perez-Soler R, Pisters KMW, Putnam JB, Jr., Richli WR, Savin M, Schrump DS, Shin DM, Shulkin A, Walsh GL, Wait J, Weill D and Waugh MKA (1999) Adenovirus-mediated p53 gene transfer in advanced non-small-cell lung cancer. *J Natl Cancer Inst* **91**: 763–771

Swisher SG, Roth JA, Komaki R, Hicks M, Ro J, Dreiling L, Yver AB, Stevens C, Putnam JB, Jr., Smythe WR, Vaporciyan AA and Walsh GL (2000) A phase II trial of adenoviral mediated p53 gene transfer (RPR/INGN 201) in conjunction with radiation therapy in patients with localized non-small cell lung cancer (NSCLC). *Proc Am Soc Clin Oncol* **19**: 461a

Tait DL, Obermiller PS, Redlin-Frazier S, Jensen RA, Welcsh P, Dann J, King M-C, Johnson DH and Holt JT (1997) A phase I trial of retroviral BRCA1sv gene therapy in ovarian cancer. *Clin Cancer Res* **3**: 1959–1968

Vasey PA, Seiden M, O'Neill V, Campo S, Johnston S, Davis J, Kirn D, Kaye SB and Shulman LN (2000) Phase I trial of intraperitoneal Onyx-015 adenovirus in patients with recurrent ovarian cancer. *Proc Am Soc Clin Oncol* **19**: 382a

Venook AP, Bergsland EK, Ring E, Nanoka-Wong S, Horowitz JA, Rybak ME and Warren RS (1998) Gene therapy of colorectal liver metastases using a recombinant adenovirus encoding WT p53 (SCH 58500) via hepatic artery infusion: a phase I study. *Proc Am Soc Clin Oncol* **1661**(17): 431a

*© 2001 Cancer Research Campaign*
